# Neurogenic Pulmonary Edema in a 22-Year-Old Male Secondary to a Ruptured Cerebellar Arteriovenous Malformation: A Case Report

**DOI:** 10.7759/cureus.117635

**Published:** 2026-10-08

**Authors:** Michael Dylan Collins, Amit Sharma, Aaron Hacker, Imani Thornton

**Affiliations:** 1 Anesthesiology, HCA Florida Westside Hospital, Plantation, USA; 2 Osteopathic Medicine, Nova Southeastern University Dr. Kiran C. Patel College Of Osteopathic Medicine, Fort Lauderdale, USA; 3 Anesthesiology and Critical Care, HCA Florida Westside Hospital, Plantation, USA

**Keywords:** acute hypoxemic respiratory failure (ahrf), arteriovenous malformations, cerebellar hemorrhage, naloxone side effects, neurogenic pulmonary edema

## Abstract

Neurogenic pulmonary edema (NPE) is an acute form of pulmonary edema that can follow a severe central nervous system insult. We describe a 22-year-old man who developed profound hypoxemic respiratory failure during presentation with a ruptured right cerebellar arteriovenous malformation (AVM). Emergency medical personnel found him unresponsive with agonal respirations after he had taken an unidentified medication for a severe headache. Because opioid toxicity was initially suspected, he received 2 milligrams (mg) of intranasal naloxone, with transient improvement in breathing. In the ED, he had pinpoint pupils, depressed consciousness, crackles, and a high oxygen requirement. After an additional 1 mg of intravenous naloxone, he became markedly hypoxemic and hypotensive, with copious pink frothy secretions, and required tracheal intubation and vasopressor support. Toxicology testing detected cannabinoids but not opioids. Computed tomography demonstrated an 18.7 milliliter (mL) right cerebellar hemorrhage, fourth-ventricular and basal-cistern mass effect, tonsillar herniation, an underlying AVM, and extensive bilateral pulmonary consolidation. An external ventricular drain was placed, but planned decompressive surgery was aborted when prone positioning caused severe oxygen desaturation and pulmonary secretions. Despite neurocritical and respiratory support, cerebral perfusion imaging later showed absent intracranial blood flow. The clinical pattern was most consistent with NPE caused by the catastrophic posterior fossa hemorrhage. Naloxone-associated pulmonary edema and aspiration were contributing considerations; the temporal deterioration after the second dose does not establish causation. This case emphasizes early recognition of pulmonary complications after acute brain injury and careful, evidence-based attribution when several mechanisms are plausible.

## Introduction

Neurogenic pulmonary edema (NPE) is characterized by the rapid onset of life-threatening pulmonary edema following acute central neurological insults. These insults include spinal cord injury, cerebrovascular disorders, traumatic brain injury (TBI), status epilepticus, meningitis, medication overdose, intracranial hemorrhage, brainstem lesions, and the postoperative period of intracranial surgery, resulting as a secondary sequelae of the inciting central nervous system (CNS) event [1]. Although increased pulmonary vascular pressure and disruption of the alveolar-capillary barrier are believed to contribute to NPE, its precise pathophysiologic mechanisms remain incompletely understood [1].

Evidence from animal studies involving antiadrenergic therapy supports a central role for intense sympathetic activation and catecholamine release in the development of NPE [2]. This sympathetic response is thought to originate in the hypothalamus and medulla after an acute CNS injury, particularly in the setting of an acute rise in intracranial pressure [3]. The resulting vasoconstriction increases systemic vascular resistance (SVR) and afterload, forcing fluid into the pulmonary circulation. In addition, venous return is increased, resulting in overload of the pulmonary circulation. Together, the direct effect is an enormous increase in pulmonary capillary pressure, damaging the alveolar-capillary barrier, therefore reducing alveolar diffusion and perfusion [3-5]. Diagnostic findings of NPE include the manifestation of pulmonary edema, pink, frothy sputum, bilateral opacities on chest X-ray, Horowitz index (PaO_2_:FiO_2_) below 200 millimeters of mercury (mmHg), acute CNS compromise with elevated intracranial pressure, resolution within 48-72 hours, and the absence of alternative causes of respiratory failure [4]. However, these findings should be considered supportive rather than absolute diagnostic criteria, particularly a PaO_2_:FiO_2_ below 200 mmHg and resolution within 48-72 hours. Management prioritizes treatment of the underlying neurologic injury and control of intracranial pressure. Hemodynamic and volume management may require vasoactive medications, diuresis, or judicious fluid administration [4,5]. When ventilatory support is necessary, a lung-protective strategy using low tidal volumes and appropriate positive end-expiratory pressure should be employed. Available evidence suggests that maintaining positive end-expiratory pressure (PEEP) below 15 cm H₂O does not significantly compromise cerebral perfusion pressure [6,7].

While NPE is commonly associated with the previously mentioned CNS insults, there are reports of pulmonary edema following administration of pharmacologic agents such as naloxone. Naloxone, an opioid receptor antagonist, is utilized as a rapid intervention to reverse opioid-induced respiratory depression. However, its use is not without consequences, as it has been implicated in the development of NPE. Naloxone-associated pulmonary edema has been reported in approximately 0.2%-3.6% of treated patients and is managed primarily with supportive respiratory care and correction of the underlying condition [8,9]. One proposed mechanism is that naloxone induces an abrupt catecholamine surge, causing pulmonary vasoconstriction, increased hydrostatic pressure, and greater capillary permeability. Together, these changes promote redistribution of intravascular fluid into the pulmonary interstitium and alveoli [9,10]. Because the mechanisms proposed for naloxone-associated pulmonary edema overlap with those of NPE, naloxone exposure may represent a competing diagnosis or potential contributor rather than an established cause of pulmonary edema in this setting.

Distinguishing NPE from other causes of acute pulmonary edema can be challenging, particularly when acute CNS injury and naloxone administration occur in close temporal proximity. Aspiration and cardiogenic pulmonary edema may produce similar clinical and radiographic findings, further complicating the diagnosis. In the present case, the patient had a ruptured cerebellar arteriovenous malformation and developed acute pulmonary edema in the setting of severe CNS injury and naloxone exposure. This case highlights the diagnostic challenge of distinguishing NPE from naloxone-associated pulmonary edema, aspiration, and cardiogenic pulmonary edema when multiple potential etiologies are present.

## Case presentation

We report the case of a 22-year-old, 173 cm, 80 kg Hispanic male (BMI, 26.7 kg/m²), who presented to the emergency department (ED) via emergency medical services (EMS) for suspected opioid overdose. EMS reported that the patient was observed taking an unknown medication and was subsequently found by his family to have developed altered mental status with agonal breathing. EMS administered intranasal naloxone 2 mg with initial observed improvement in respiratory status. Past medical history was significant for non-insulin-dependent diabetes mellitus (NIDDM), asthma, recreational marijuana use and gastroesophageal reflux disease. EMS reported active home medications to include famotidine, pantoprazole, and ondansetron.

Physical examination was significant for bilateral pinpoint pupils without accommodation, and the patient was unable to follow commands. Initial National Institutes of Health Stroke Scale (NIHSS) and Glasgow Coma Scale (GCS) were 40 and 8, respectively. Notable physical exam findings included normal S1/S2, normal capillary refill, coarse lung sounds with crackles, a soft and non-tender abdomen, and extremities without edema, clubbing, or cyanosis. Initial vital signs revealed: blood pressure (BP): 106/77mmHg, heart rate: 83 beats per min (bpm), respiratory rate: 20 breaths per minute, pulse oximetry: 95% on 15 liters/minute (L/min) via non-rebreather mask with FiO_2_ 100%. Following administration of an additional dose of intravenous (IV) naloxone 1 mg, the patient became hypotensive with a BP of 79/45 mmHg, tachycardic with a heart rate of 122 bpm, and with decreased oxygen saturation via pulse oximetry of less than 60%. Pink, frothy discharge from the oropharynx was noted. Re-assessment revealed a new GCS of 5 as evidenced by non-purposeful movement of bilateral upper and lower extremities, nonspecific, incomprehensible sounds, and eye opening only to pain. Rapid sequence intubation (RSI) was initiated with a 7.5mm endotracheal tube via video assistance. A 20-gauge right radial arterial line and 16 cm triple-lumen right internal jugular vein central venous catheter were placed for hemodynamic monitoring and vasopressor support initiation, respectively. Norepinephrine infusion was titrated to a mean arterial pressure (MAP) goal of 65mmHg or greater at 0.04 micrograms per kilogram per minute (mcg/kg/min), while 2 liters of crystalloids were bolused. Initial arterial blood gas (ABG) at that time was as follows: pH: 7.08, pCO_2_: 55, PO_2_: 58.7, HCO_3_: 15.8 (Table 1). The toxicology report was positive only for cannabinoids. Other notable labs on arrival include WBC: 25.8, K+: 3.3, glucose: 307, and lactic acid: 8.2 (Table 2).

**Table 1:** ABG results taken on hospital day 1 (in the ED) and on hospital days 2, 3 and 4 (in the ICU) pH: potential of hydrogen; PaCO2: partial pressure of carbon dioxide; PaO2: partial pressure of oxygen; HCO3: bicarbonate; O2Sat: oxygen saturation; ABG: Arterial blood gas

ABG Lab Value (units)	Hospital Day 1 (in the ED) 14:30	Hospital Day 1 (intubated in the ED) 15:30	Hospital Day 2 (in the ICU)	Hospital Day 3 (in the ICU)	Hospital Day 4 (in the ICU)	Reference Range
pH	7.08	7.06	7.23	7.47	7.3	7.35-7.45
PaCO_2_	55	78.2	40.9	33.1	43.8	34-45 mmHg
PaO_2_	58.7	117.2	115.2	118.5	52.5	80-100 mmHg
HCO_3_^-^	15.8	21.6	16.9	23.4	21.1	22-26 mEq/L
O_2_Sat	95	58	99	98	84	95-100%

**Table 2:** CMP and CBC results taken on hospital day 1 (in the ED) and on hospital days 2, 3 and 4 (in the ICU). BUN: Blood urea nitrogen; eGFR: Estimated glomerular filtration rate; AST: Aspartate aminotransferase; ALT: Alanine aminotransferase; WBC: White blood cells; RBC: Red blood cells; HGB: Hemoglobin; HCT: Hematocrit; MCV: Mean corpuscular volume; MCHC: Mean corpuscular hemoglobin concentration; RDW: Red cell distribution width; PLT: Platelet; MPV: Mean platelet volume; L: Low; H: High; HC: Critical high; CMP: Comprehensive metabolic panel; CBC: Complete blood count; ED: Emergency department; ICU: Intensive care unit.

CMP Lab Value (units)	Hospital Day 1 (in the ED) 14:30	Hospital Day 1 (intubated in the ED) 15:30	Hospital Day 2 (in the ICU)	Hospital Day 3 (in the ICU)	Hospital Day 4 (in the ICU)	Reference Range
Sodium (mmol/L)	139	143	163	161	154	135-145
Potassium (mmol/L)	3.3	5.6	3.8	3.6	3.8	3.5-5.2
Chloride (mmol/L)	107	104	134	133	127	95-110
Carbon Dioxide (mmol/L)	15	27	17	26	24	19-34
BUN (mg/dl)	17	20	14	15	21	6-33
Creatinine (mg/dL)	1.11	0.91	1.29	1.35	3.05	0.43-1.13
eGFR (non-AA) (ml/min/L)	>60	>60	>60	40	26	>60
Lactic Acid (mmol/L)	8.2	3.5	4.7	7.1	2.3	0.4-2.0
Total Bilirubin (mg/dl)	0.4	0.7	0.8	1.6	2.0	0.1-1.2
AST (units/L)	45	56	100	133	129	10-40
ALT (units/L)	57	49	68	35	38	10-60
Total Alk Phosphatase (units/L)	94	83	68	76	102	20-130
Creatinine Kinase (units/L)	2741	N/A	N/A	297	N/A	39-308
CBC						
WBC (cells per 10^3/uL)	25.8	24.1	30.6	19.1	21.7	4.0-10.5
RBC (cells per 10^6/uL)	5.87	5.3	5.46	3.83	4.04	4.63-6.08
HGB (g/dL)	16.4	15	10.9	11.4	10	13.7-17.5
HCT (%)	48.7	44.7	32	34.3	29.8	40.1-51.0
MCV (fl)	83	84.3	83.7	83.6	84.7	79.0-92.2
MCHC (g/dL)	33.7	33.6	33.8	34.1	33.6	32.3-36.5
RDW (%)	12.9	13.1	13.4	13.2	14.1	11.6-14.4
PTL count (cells per 10^3/uL)	307	256	262	144	83	150-400
MPV (fl)	11.9	11.5	11.9	11.3	12.2	9.4-12.4
Absolute neutrophils (cells 10^3/uL)	20.59	22.37	28.59	20.47	21.9	1.56-6.13

Upon re-evaluation two hours later in the ED, the patient was noted to have a dilated/unreactive right pupil with a less dilated, sluggish left pupil, coarse breath sounds with crackles bilaterally, unresponsiveness to noxious stimuli, and absent cough and gag reflexes. Shortly after admission, the family arrived in the ED and stated that the patient had the worst headache of his life prior to arrival. He was emergently taken for a computed tomography angiography (CTA) scan and found to have a right cerebellar hemorrhage with a volume of 18.7 mL, with mass effect on the fourth ventricle and basilar cisterns, as well as inferior tonsillar herniation (Figure 1A-1B). An arteriovenous malformation (AVM) in the right cerebellum in the hemorrhagic region was also identified as the source of hemorrhage. Chest CT (Computed Tomography) was remarkable for extensive bilateral lung foci of consolidation consistent with pulmonary edema, along with contrast retention within the renal cortex of the kidneys, which can be seen in shock (Figure 1C). Given the acute CNS injury and the temporal relationship between the neurologic deterioration and pulmonary findings, neurogenic pulmonary edema was highest on the differential; however, aspiration, naloxone-associated pulmonary edema, and cardiogenic pulmonary were also considered. Interventional Neurology, Neurosurgery, and ICU were consulted. Interventional Neurology planned for an embolization the following morning, while Neurosurgery placed an external ventricular drain with a plan for emergent suboccipital craniotomy. The patient was given IV mannitol, 3% normal saline, levetiracetam, piperacillin/tazobactam, and famotidine.

In the operating room, the patient was placed in the prone position and immediately became difficult to ventilate, with a significant amount of frothy, pink fluid exuding from the endotracheal tube. His peripheral capillary oxygen saturation (SpO_2_) dropped to 64%. He was quickly returned to a supine position, and the anesthesia team performed a bronchoscopy, which confirmed severe pulmonary edema. Due to unstable vitals and the high probability of mortality from being in the prone position for 3-4 hours, the decision was made to abort the procedure in favor of medical management in the interim care unit for intracranial hypertension, as well as continuing the external ventricular drain.

In the ICU, the patient was sedated and continued to remain on ventilatory support. He developed acute renal failure and heart failure (ejection fraction (EF) 10% on echocardiogram) secondary to shock and was started on IV furosemide with minimal improvement in urine output. Repeat CT brain two days status post AVM rupture showed decreased cerebellar hematoma, decompressed ventricular system, and no new hemorrhages (Figure 1D-1E). He was then weaned off sedation and remained unresponsive to stimuli. A nuclear medicine brain flow study was completed and revealed no intracerebral blood flow consistent with brain death (Figure 1F). After the brain flow study, the patient was pronounced deceased three days status post AVM rupture.

**Figure 1:**
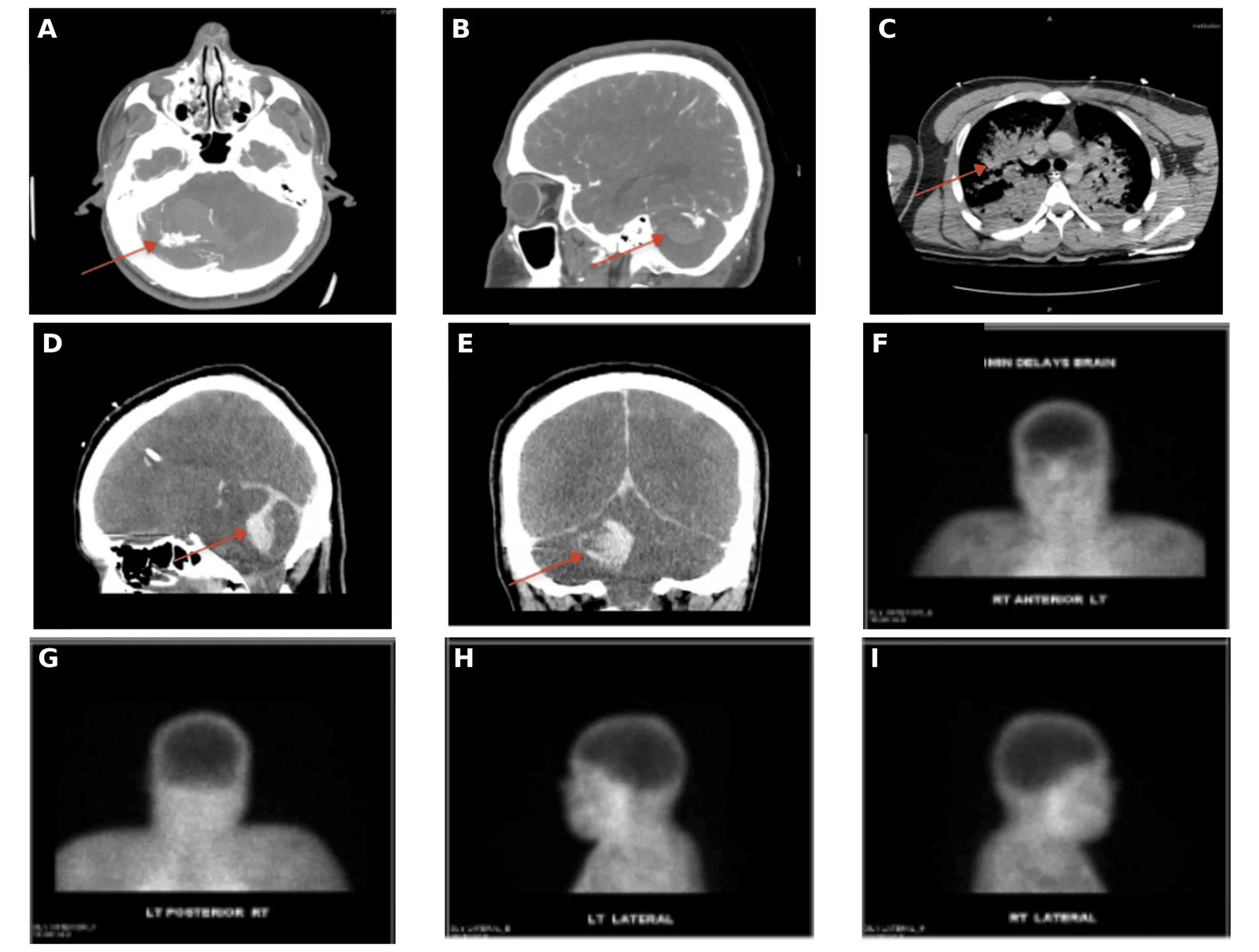
Neuroimaging, chest imaging, and cerebral perfusion study. (A) Axial and (B) Sagittal CT angiographic images show the right cerebellar hemorrhage and associated vascular abnormality; red arrows indicate the hemorrhagic AVM region. (C) Axial chest CT shows extensive bilateral air-space consolidation; the red arrow identifies representative confluent opacity. Follow-up: (D) Sagittal and (E) Coronal head CT images show decreased hematoma size two days after rupture; red arrows identify the residual posterior fossa hematoma. Radionuclide cerebral perfusion images in (F) Anterior, (G) Posterior, (H) Left lateral, and (I) Right lateral projections show no intracranial radiotracer activity. AVM: Arteriovenous malformation

## Discussion

NPE arises from the dysregulation of sympathetic nervous system activity, resulting in a cascade of events leading to increased pulmonary vascular permeability and subsequent alveolar flooding. The surge in catecholamine release and sympathetic outflow triggers a series of hemodynamic changes, including elevated systemic and pulmonary pressures, capillary leakage, and impaired alveolar fluid clearance. These alterations collectively contribute to the development of pulmonary edema and may occur with or without primary cardiac dysfunction [1-3]. Similar to neurologic insults, rapid administration of Naloxone leads to an abrupt surge in sympathetic activity, resulting in a cascade of events that contribute to increased pulmonary capillary permeability, elevated hydrostatic pressure, and ultimately, the development of pulmonary edema. In the present case, the temporal association between the second dose of naloxone and subsequent respiratory deterioration raises the possibility of a contribution from naloxone-associated pulmonary edema; however, this association does not establish causality.

Neurogenic pulmonary edema can arise from diverse etiologies, including traumatic brain injury, subarachnoid hemorrhage, seizures, and neurosurgical procedures. The incidence of NPE varies depending on the underlying neurological insult and the population studied. Reports suggest that NPE occurs in approximately 6% to 25% of patients with severe traumatic brain injury [4,5]. Additionally, cerebellar hemorrhage, particularly aneurysmal rupture, is a well-recognized trigger for NPE, with an incidence ranging from 10% to 30% [6]. Pulmonary edema following naloxone administration has gained attention in recent years, prompting further investigation into the underlying mechanisms and pathophysiology of this phenomenon. Although this complication appears to be rare, it has been reported in patients with co-existing heart disease, obstructive sleep apnea, and in young adults who received standard doses of naloxone [7]. While NPE has been reported in cases of opioid overdose, there is limited evidence to suggest that naloxone itself directly causes NPE in patients without opiate use, as is the case of our patient [8]. The effects of naloxone administration in patients without narcotics is a topic that requires further investigation.

The management of neurogenic pulmonary edema necessitates a multidisciplinary approach involving neurologists, intensivists, and anesthesiologists. The primary goal is to optimize neurological outcomes while simultaneously addressing the potentially life-threatening respiratory consequences of NPE. Treatment strategies often involve supportive measures, such as mechanical ventilation and judicious fluid management, along with addressing the underlying neurological insult. The choice of therapeutic interventions depends on the severity of the pulmonary edema and the overall clinical condition of the patient. Diuretics, inotropic agents, and vasodilators may be considered, but caution is warranted to prevent exacerbating hypovolemia [9].

Anesthetic management in patients with neurogenic pulmonary edema requires careful consideration of the underlying neurological condition, cardiovascular stability, and respiratory status. Maintaining optimal oxygenation, avoiding excessive positive pressure ventilation, and meticulous attention to hemodynamic stability are crucial aspects of anesthetic care. Additionally, close monitoring of intracranial pressure and cerebral perfusion is essential to prevent exacerbation of the underlying neurological insult. The choice of anesthetic agents, including induction agents and analgesics, should be tailored to minimize adverse effects on cerebral hemodynamics. In the present case, the patient's severe pulmonary edema and hemodynamic instability limited the ability to proceed with the planned surgical intervention and required continued medical management in the intensive care unit.

Neurogenic pulmonary edema presents a challenging clinical scenario requiring prompt recognition and targeted management. Despite its relatively low incidence, NPE can have severe consequences, necessitating a comprehensive understanding of the underlying pathophysiology and adherence to current guidelines. Additionally, the incidence and pathophysiology of NPE in opiate users and opiate naïve patients given Naloxone requires further investigation. In this case, the acute neurologic injury and temporal relationship between the cerebellar hemorrhage and pulmonary edema support NPE as a likely explanation; however, the presence of severe cardiac dysfunction, the temporal relationship to naloxone administration, and the possibility of aspiration prevent definitive attribution to a single mechanism. Collaborative efforts among neurologists, intensivists, and anesthesiologists are essential to optimize patient outcomes and mitigate the potential complications associated with this complex syndrome.

## Conclusions

This case report describes the presentation of severe pulmonary edema most consistent with neurogenic pulmonary edema in the setting of a cerebellar hemorrhage and administration of naloxone in a 22-year-old male patient in the setting of a ruptured arteriovenous malformation. Even though the patient was medically managed with mechanical ventilation, aggressive fluid restriction, and hemodynamic monitoring, his condition ultimately proved to be fatal. The results of our case presentation underscore the importance of maintaining a high index of suspicion for neurogenic pulmonary edema, particularly in patients presenting with neurological compromise. The neurologic injury was the most plausible trigger for NPE, although other contributing mechanisms could not be excluded. Although respiratory deterioration followed a second naloxone dose, pulmonary findings were present beforehand and causation cannot be inferred. Clinicians should recognize pulmonary edema early after acute brain injury, consider aspiration and cardiac dysfunction in the differential diagnosis, and balance lung-protective ventilation with preservation of cerebral perfusion. In this setting, naloxone exposure, aspiration, and myocardial dysfunction may also contribute to the development of pulmonary edema and should be considered when determining the underlying cause.
